# Reflections on “Orchestrating for Impact”: Harmonizing across Stakeholders to Accelerate Global Health Gains

**DOI:** 10.4269/ajtmh.21-1101

**Published:** 2022-03-15

**Authors:** Julie Jacobson, Alan Brooks

**Affiliations:** ^1^ Bridges to Development, Vashon, Washington;; ^2^ Bridges to Development, Geneva, Switzerland

## Abstract

For developing countries and partners to accelerate the availability of new innovations to communities in need of intervention, it is necessary to find improved ways to work together that challenge today’s status quo in global health. By adopting new approaches and accepting shared risk and reward, diverse partners can work together to accelerate progress toward a common global health goal. A symphony orchestra—with a conductor who recruits, arranges, harmonizes, and orchestrates diverse musicians to work together to create something not possible by individual musicians on their own—provides an apt metaphor for successful global health initiatives. In such settings, stakeholders must be engaged and harmonized, workplans composed, and partnerships conducted such that all stakeholder roles progress from workplans that are sequential and independent to those that are integrated and interactive. Reflecting on the successful global health partnerships discussed here and beyond, we use this orchestral metaphor to illustrate the elements needed to make partnerships increasingly successful.

## INTRODUCTION

Development and deployment of new health products for low- and middle-income countries rely on a complex series of decisions and actions made by multiple independent groups of stakeholders working in very different settings around the world. Despite this complexity, however, the past several decades have seen great successes achieved through the use of product development partnerships and public private partnerships to create new models for working together. Indeed, we now have the opportunity to build off these experiences to look at new ways to optimize and improve product development and deployment for public health needs.

We document the experiences of four recent global health partnerships that have accelerated achievement of their global health targets significantly, ^1^^–^ ^4^ and thereby provided an emerging profile of the needs and demands for developing a new product and/or strategy and integrating it successfully into health systems. This profile includes not only the steps of the process, but also how the work is done and which types of partnerships are required. Reflecting on these experiences and on the elements of initiation, planning, and execution described extensively in the gray and published literature, ^5^ we recognize that the *art* of successful partnership aligns well with the metaphor of an orchestra performing a symphony. Each instrument plays a critical role that is unique, and a symphony is only possible when each of those instruments is working closely together with a shared goal and an understanding of the specific contributions of the others. There are three steps in this metaphorical process to help future partnerships be successful: 1) *building the orchestra:* fine-tuning the team, 2) *composing the score:* integrating work plans, and 3) *conducting the symphony:* working in partnership.

## BUILDING THE ORCHESTRA: FINE-TUNING THE TEAM

The first step in building the partnership team is understanding who is needed to achieve the goal of the partnership. Who needs to make the decision to act, to get others to act, or to support the work enough to enable it to happen? Global health is complex, and there are multiple stakeholders at various levels, including countries and communities with endemic disease, civil society organizations, implementing partners (e.g., nongovernmental organizations), donors, policymakers (e.g., the WHO), regulatory bodies, academic researchers, and the private sector (e.g., pharmaceutical companies). Like every instrument in an orchestra, each of these stakeholders has something special to contribute. Each has a unique perspective, understanding of risks, and set of incentives—institutional, professional, or personal—that drive them to act based on the information they receive and the system within which they work. To understand their needs and motivations for achieving the goal, a stakeholder analysis should be conducted to define the following: the assets each stakeholder brings, the role each stakeholder has in achieving the goal, the data each stakeholder needs to know, the perceived risks and incentives held by each stakeholder, and the timing that is optimal for each stakeholder to be able to engage effectively.

Particularly important among global health stakeholders are the end users and populations at risk for a disease. These are the people who will ultimately receive and then choose to accept, reject, or, ideally, demand the intervention—the audience in our metaphor. If the audience does not come or does not like what is offered, the efforts will be in vain. Many global health initiatives and products have failed to reach their potential by undervaluing or ignoring the end users’ perspective until it was too late. These stakeholders may not always be present in the initial meetings, but their input must be included in the prioritized work plan and intervention design from the beginning. Engaging them early will help to identify and understand their preferences, fears, desires, and knowledge of how this intervention will “play to the crowd,” by fitting into their lives, community, and work. It is important to discuss with these communities their essential role in the pathway to achieve the desired impact, and to recognize and honor their contributions.

## COMPOSING THE SCORE: INTEGRATING WORK PLANS

After understanding the stakeholders and their needs, an integrated work plan is then composed—just as bringing together the music for individual instruments creates a symphony’s single score. To ensure harmony, a shared set of principles needs to be developed as part of creating an integrated work plan. These principles should define the expectations of the stakeholders, lay out or describe how work will be done, and support a communications plan among partners for information sharing and timely, efficient use of results. The guiding principles—formal or informal—help define how the data that stakeholders need to take action can be generated, including the ethical standards and any procedures unique for each stakeholder’s workplace or environment. For some stakeholders, the input required may already be a priority of their organization and is well aligned with the activities of the partnership. For others, when what is needed is tangential to their usual work, the stakeholder’s commitment may be more challenging and may require additional partner input.

Developing a project’s overall work plan requires review and understanding of the components needed to achieve the goal, and a plan for each stakeholder’s work stream. With this information, a work plan can be created with overlapping work streams operating in parallel, with activities starting as early as possible *whenever possible*. Such a plan requires both explicit identification of partner interdependencies and the harmonization of activities to define who relies on whom to take action. To develop this plan, it is helpful to work backward, starting from the desired goal and working back to current activities. When all the process elements are outlined, one can then identify the potential rate-limiting steps to achieving the necessary stakeholder action. Defining potential roadblocks allows stakeholders to develop strategies to address them early, potentially avoiding negative outcomes such as delayed decisions or decisions not in line with the overall plan.

Identifying the *shortest timeline* relies on knowing when sufficient data will be available to trigger a decision, recognizing the varied needs and processes that different stakeholders must follow in their work. A common rate-limiting step is the need for peer-reviewed publication. If partners must wait for publication to act on data, there can be significant delays in action. On the other hand, action can be triggered by data *prior* to publication, if a process is in place that is aligned with principles and agreed in advance so that scientific rigor and independence of data review and analysis can be ensured. In the development and deployment of triple-drug therapy for lymphatic filariasis, because the researchers had already established a pathway *accepted by the WHO* and in line with the drug donation commitments from Merck & Co., Inc., five countries had already introduced the treatment into their national programs prior to the publication of data from even the first randomized controlled trial, speeding up the development process significantly. ^1^ Each activity in the shared work plan should be mapped back to how it helps achieve the shared goal and how it enables actions to be taken by the stakeholders. Although there might be a lot of interesting science done in these activities, all undertaken with good intentions, if an activity does not contribute to the end goal, it can slow progress and should not be included the work plan.

## CONDUCTING THE SYMPHONY: WORKING IN PARTNERSHIP

It is unlikely that a single stakeholder will have end-to-end control of any project, so there is a critical role for a conductor. The conductor is an individual, team, or organization that coordinates the stakeholders to ensure they work together as efficiently and effectively as possible toward the shared goal. The entity taking on this role must earn and maintain trust with all stakeholders, and must establish good communications that allow for monitoring across all workstreams and timelines, adjusting for interdependent shifts. The conductor needs to understand how each workstream is interrelated, the importance of timing and transition, and how to support the stakeholders in their work. If one stakeholder is held back unexpectedly, others need to adapt. The conductor should work preemptively to identify where there are gaps or where a stakeholder might be overwhelmed and need more help or encouragement. The symphony relies on commitment and incentives focused on the shared goal to maintain partner alignment, motivation, and cooperation.

An effective partnership must be flexible enough to acknowledge and attend to stakeholder needs that change, evolve, or shift over time. Although trust and communication are essential, this does not mean that everyone must know everything at all times. Information overload, especially with details that are unimportant to a specific partner or to wider stakeholders, can actually decrease effective communication, and important details can get lost. Every member of the orchestra does not need to recognize every note played across all instruments. To get the communication element right, one must identify what needs to be communicated, to whom, by whom, officially and unofficially, with the right frequency and timing, and with the changes needed over time. Because of the diversity of partners working together, there are varying levels of risk involved for each. It is important for partners to identify, discuss, share, and accept risk. Acknowledging and managing risks will rely on good communication across the partners and with the broader set of stakeholders.

As the project moves forward and data emerge, underlying assumptions should be reevaluated to determine whether they are all still relevant. Successes, as well as setbacks, should be shared so that collaborative problem solving and risk mitigation can be done across all partners as appropriate. For all of this organization and assessment, the essential role of the conductor must ensure that the work being done remains in line with the founding principles put forward by the partners and is in harmony with the shared goal.

## CONCLUSION

**Figure 1.**
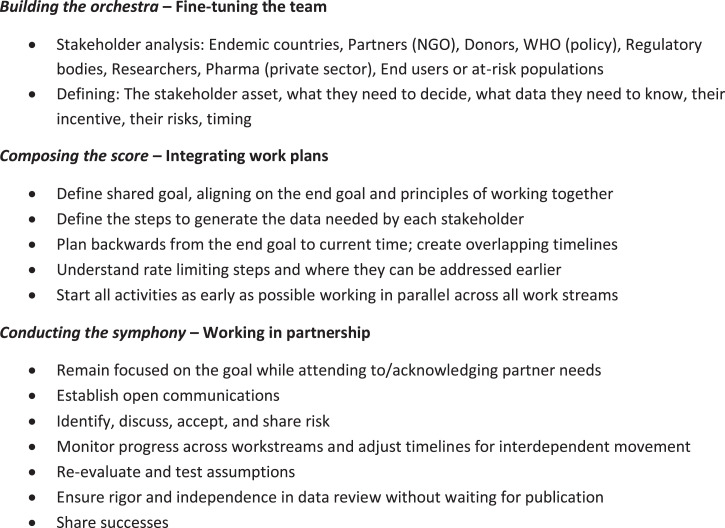
Key steps in orchestrating for impact. NGO = nongovernmental organization.

Accelerating global health research gains requires changing our orientation and shifting our goals from just completing the next study or securing the next grant to focusing on the impact we are trying to achieve. For those of us working in development, and especially those of us creating new health interventions, changing our perspective from individual studies to how we fit into the larger landscape and how we contribute to a more ambitious goal can result in a fundamental shift with a dramatic effect. It reflects the difference between a group of musicians playing their own songs and a symphonic orchestra performing with musicians working together to build on each instrument’s strengths and each musician’s unique abilities to achieve something much greater than would ever be possible alone. If multiple partners are committed to a shared goal, such as developing and scaling up a new medicine to treat neglected diseases, this symphonic approach holds real potential for diverse contributors to tackle a global health problem jointly and to achieve a mutually shared goal. The three elements—*building the orchestra*, *composing the score*, and *conducting the symphony*—in the orchestral metaphor are translated into their global health counterparts in Figure 1. Perhaps envisioning the harmony and teamwork of a symphony can help us see beyond spreadsheets, calendars, and deadlines to see the beauty, challenge, and elegance of a well-functioning partnership that can serve those at-risk both more efficiently and with more joy.
